# On-farm diversity and production challenges in Ethiopian tef [*Eragrostis tef* ((Zuccagni) Trotter)] landraces from Arsi zone, Ethiopia: Implications for breeding and conservation

**DOI:** 10.1016/j.heliyon.2025.e41837

**Published:** 2025-01-10

**Authors:** Fekadu Gadissa, Muhamed Adem

**Affiliations:** a Madda Walabu University, College of Natural and Computational Sciences, Biology Department, P.O.Box 247, Bale Robe, Ethiopia; b Madda Walabu University, College of Agriculture, Forestry Department, P.O.Box 247, Bale Robe, Ethiopia

**Keywords:** *Eragrostis tef*, Genetic erosion, Tef landraces, Utilization, Production

## Abstract

**Context:**

Tef [*Eragrostis tef* ((Zucc.) Trotter)] is a remarkable indigenous crop, highly adaptive and resilient to erratic and extreme climatic and soil conditions. It is a major staple food in Ethiopia and is usually cultivated for household consumption and the generation of income. However, nowadays, the crop particularly, the landraces are exposed to genetic erosion owing to biotic and abiotic factors. Thus, detecting the current status and production bottlenecks of the crop is key to enhance its production and conservation.

**Objective:**

The main objectives of this study were to assess the extent of on-farm diversity, genetic erosion and current production challenges of Tef landraces in Arsi Zone, Oromia region, Ethiopia.

**Methods:**

The study was conducted in the Arsi zone, Southeast Ethiopia using 400 selected farmers. Data were collected using a semi-structured questionnaire and analyzed using Minitab version 19. The respondent farmers included the different sex groups, age groups, educational status, marital status, and religious outlooks.

**Results and conclusion:**

*S*: A total of 26 Tef landraces have been identified to be cultivated over the last 2 to 3 decades and/or still in production in the study areas. The respondents indicated differences in the landraces with regard to morphological features which along with the naming's could suggest their genetic distinctiveness. Patterns of on-farm diversity in the landraces showed a varied abundance in the area where eleven landraces showed a relatively higher estimate (*D* = 0.75 in Sergegna to *D* = 0.58 in Bulga). With the exception of Aruso Tef (*D* = 0.47), all the remaining landraces showed minimal or no (*D* = 0.00) genetic abundance. The extent of the Sorenson similarity index revealed a higher similarity (0.69 in Enkelo Wabe *vs* Arsi Robe to 0.77 in Shirka *vs* Arsi Robe) and the areas revealed a higher landrace richness (R) and evenness index (E). However, patterns of temporal genetic diversity and extent of genetic erosion revealed that only 10 are grown currently (combined genetic erosion of 61.54 %). In general, biotic and abiotic factors are challenging the current production of the crop in the area. Hence, researchers and all stakeholders should pay attention to conserving and breeding this important food crop.

**Significance:**

Understanding the extents of on-farm diversity and possible production challenges of the crop could facilitate and enhance its breeding, conservation and utilizations and hence, contribute to the food security of the country.

## Highlights

• Significant number of Tef landraces have been identified from Arsi zone.
• The landraces bear good estimate of on-farm diversity.
• The higher extent of combined genetic erosion in the area needs special attention.
• Current production of Tef landraces in the area is being affected by biotic and abiotic factors.
• Special conservation and breeding actions are needed to keep and enhance its productivity.

## Graphical abstract

**Figure:**
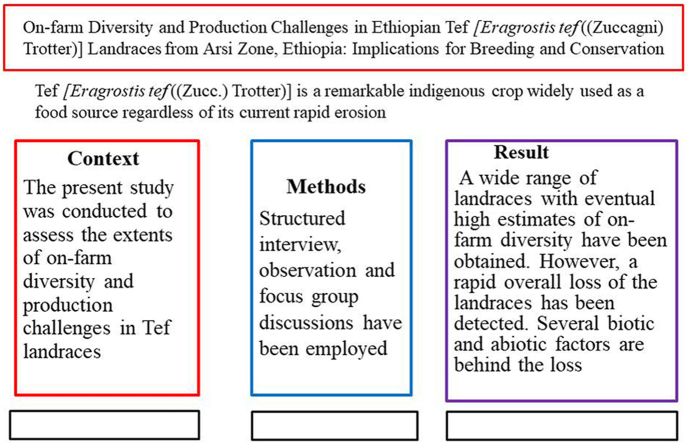

## Abbreviations

ANOVA: Analysis of Variance
GE: Genetic Erosion
GI: Genetic Integrity
QSAE: Quality Standard Authority of Ethiopia
CSA: Ethiopian Central Statistical Authority
DAs: Development Agents

## 1 Introduction

Tef [*Eragrotis tef* (Zucc.) Trotter] is the only cultivated species of the genus *Eragrotis* and was domesticated by the pre-Semantic inhabitants of the Ethiopian highlands around 4000 BC. It is one of the country's most important annual food crops grown by over six million subsistence farmers and provides staple food for about 70 million Ethiopians [1]. It is considered as an excellent source of essential amino acids, especially lysine, which is often limiting in most cereal foods and is preferred for its being gluten-free [2]. As a result, it has gained worldwide popularity and serves as an alternative cereal for people with gluten sensitivity and coeliacs. It also has high fiber content and low glycemic index, which is important for diabetic people, and its high iron content also prevents anemia [3].

Compared to other indigenous cereals, Tef has remarkable resilience to marginal land and bear broad adaptive potential to the heavy, waterlogged clay soil conditions of the Ethiopian highlands, where population pressure and land degradation are major problems [4]. Therefore, it is considered as a food security crop for most Ethiopian subsistence farmers. In addition, as Ethiopia is the center of origin for this crop, most of the country's Tef genetic resources are in the form of landraces, so they can be expected to have a high degree of genetic diversity and adaptability to changing environmental and ecological conditions. Therefore, they are expected to provide a reliable basis for improving food security and developing crop diversification in the country's moisture-stressed and challenging agro-ecological areas [4].

However, in recent years, the existing Tef landraces are being neglected and rapidly eroding from several areas where they were previously cultivated. Several biotic and abiotic factors are contributing to their rapid genetic loss [5]. One of the factors include the current erratic climate condition which is projected to become more variable in the years to come due to several factors such as human population increase, rapid deforestation, fossil fuel-based industrializations, extensive use of improved varieties, etc. Wide-scale use of traditional and unimproved cultural practices is also another yield limiting factors and aggravated the loss of some landraces [6].

Such loss of landraces and eventual genetic diversity in a specific area over a given period of time is known as genetic erosion. It includes the loss of a single gene or specific gene combinations that are expressed in landraces or varieties. Therefore, it is a result of how genetic diversity changes over time [7]. It has led to a loss of variability at the crop, variety, or allele level and is the primary threat to landraces [8,9]. It lowers the crop's evenness and richness and may eventually cause the total loss of a given germplasm [10].

Several approaches have been employed to estimate the extent of on-farm diversity and the degree of genetic erosion that a particular taxon faces in a certain region over a given time. One of the approaches is applying on-farm diversity indices such as spatial diversity, landrace evenness, landrace richness and estimating the extent of genetic erosion directly or indirectly from the historical and existing situation. Assessing the possible and potential production challenges is also important to understand the underlying causes and thus, hints the proper measures to be taken to reverse the loss. Thus, the present study was initiated to establish baseline information on the extent of on-farm diversity, genetic erosion and production challenges in Tef landraces from Arsi zone, Southeast Ethiopia. The information generated could be used for further breeding and genetic conservation of the crop.

## 2 Research methodology

### 2.1 Description of the study area

**Fig. 1:**
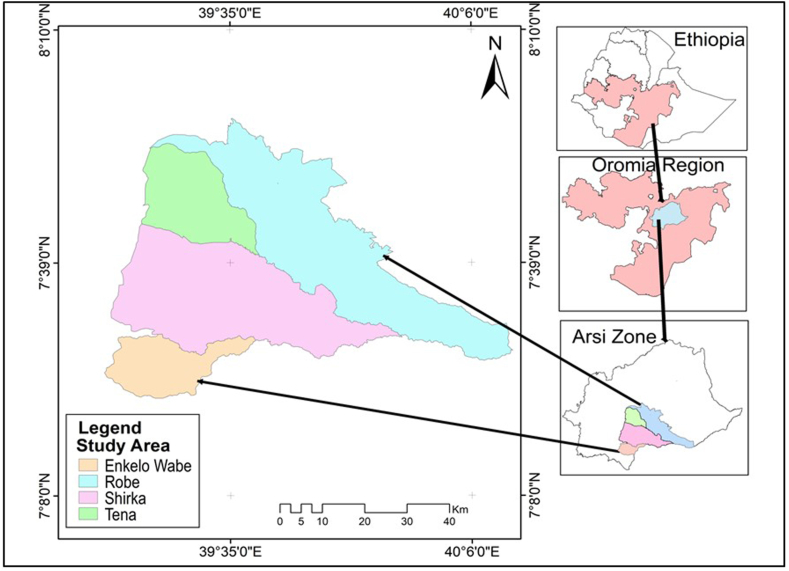
Map of the study area showing Ethiopia, Oromia, Arsi zone and the study districts. The map was original and constructed using geographic coordinates and elevation data gathered from each collection sites using global positioning system (GPS); Robe refers to Arsi Robe.

The study was conducted in four selected districts (Shirka, Tena, Arsi Robe, and Enkelo Wabe) in Arsi Zone (Fig. 1), which are situated in the Southeast direction of Addis Ababa, the capital of Ethiopia. The districts lie between 7°08′18″ to 7°28′08’’N (latitude) and 39°16′36″ to 39°53′18″ E (longitude). The area has an altitudinal range of 2220 mm–2290 m.a.s.l. and an annual average maximum and minimum temperature of 23.2 °C and 10.5 °C respectively, and receives an average annual rainfall of 823 mm (source: *Arsi Zone Agricultural* and Natural Resource *Office (AZANRO), (2023)*

The districts were selected on the basis of their potential and suitability for Tef landraces growing; the recent shift towards favoring improved Tef varieties and wheat, and less uniformity in socio-cultural and religious outlooks of the farmers that posed some pressure on the management practices and selection of landraces for their end-use qualities.

### 2.2 Framework of thoughts

Regardless of being the center of origin and diversity for several crop species, Ethiopia is among the top vulnerable countries to the rapid loss of genetic resources and has very scarce detailed baseline data on the current status of indigenous and endemic crops. Moreover, the country has no well-documented information regarding the extent of on-farm genetic diversity, production constraints, and opportunities of conserving indigenous food crops and their wild relatives [11] except few such as barley landraces [12]. One of the driving forces for the shortage of such information is the locally restricted uses of the crops and the current national and research focus of the country that largely targets commercial and high-yielding crops such as wheat, barley especially malt barley, coffee, and commercial fruits and vegetables. Tef landrace is one of the indigenous food crops with little baseline information and no standard methodology. Thus, the current study was aimed at assessing the extent of on-farm diversity and production challenges in the crop using the local indigenous knowledge and practices of farmers who have been engaged in the farming practices for a couple of decades.

The research was designed to firmly exploit farmers' traditional knowledge and the genetic distinctiveness of landraces to estimate the extent of on-farm diversity and genetic erosion in the crop following the protocols and models by Refs. [13,14]. The models were long been used as a predictor of variability based on all of the necessary confirmations and assumptions, with the goal of minimizing any discrepancies between the names assigned to landraces and their genetic distinctiveness. Some of the assumptions included the crop's self-pollinating nature, which plays an important role in preserving genetic integrity over long periods of time. Similarly, uniformity among larger members of the community in terms of ethnicity, socioeconomic, and sociocultural background was considered as an input to reduce the problem of naming the same landrace with different names. In addition, given that the information is gathered from traditional farmers and best explains the selection, management, maintenance, and preservation of landraces, the research may, in general, compromise the issues associated with the lack of a well-developed and standardized methodology for determining the degree of on-farm diversity and genetic erosion in crop species. The uniformity of the farming system and cropping patterns was also regarded as an important factor in avoiding the use of similar names for landraces with different morphological and physiological characteristics.

In general, the research could compromise the challenges related to the lack of standardized and well-developed methodology for determining the degree of on-farm genetic diversity and genetic erosion in crop species as the information is gathered from traditional farmer and best explains the choice, management, utilization, and preservation of landraces.

### 2.3 Study design and sample size

A community-based cross-sectional research design was used with a focus on selected farmers' districts and kebeles (the smallest administrative setup) within districts that were identified through a quick preliminary informal survey and discussions with zonal and district agricultural bureau experts. Data were gathered from both primary and secondary sources during 2023/24.

The sample size was determined using a standard formula suggested by Ref. [15] considering a 95 % confidence level and an error margin of less than 10 % as proposed by Ref. [16] (Table 1).

**Table 1:** Administrative districts, kebeles and number of household heads (respondents) that were sampled for the study.

No	Study Districts	Study Kebeles	Number of HHs per Kebele			Total sample farmers		
			M	F	Sum	M	F	Sum
1	*Shirka*	Tereta	1238	77	1315	59	4	63
		Hella Zanbaba	853	55	908	41	3	44
		**Sum**	**2091**	**132**	**2223**	**100**	**7**	**107**
2	*Tana*	Sole Chafa	753	46	799	36	2	38
		Bakaksa	1016	38	1054	49	2	51
		**Sum**	**1769**	**84**	**1853**	**85**	**4**	**89**
3	Arsi Robe	Sabro Chafe	1056	30	1086	51	1	52
		Ataba Robe	984	48	1032	47	2	49
		**Sum**	**2040**	**78**	**2118**	**98**	**3**	**101**
4	Enkelo Wabe	Bokoji Mirte	763	36	799	37	2	39
		Machitu Goto	1212	132	1344	58	6	64
		**Sum**	**1975**	**168**	**2143**	**95**	**8**	**103**
		***Sum total***	***7875***	***462***	***8337***	***378***	***22***	***400***

Accordingly, $N=\frac{0.25}{{SE}^{2}}$ = 400 Where, N = sample size; SE (standard error) = 2.5 %

To achieve equal coverage and to obtain a relatively balanced response and conclusion, the study districts were assigned a fairly equal number of respondents using the formula **n×P**_**i**_ where; n is the sample size selected from eight sample kebeles, in this case 400 head of households, P_i_ is the proportion of household population included from the stratum or each kebele divided by the total population size of the kebeles which is 8337 (Table 1).

### 2.4 Data collection methods

A variety of efficient data collection techniques such as focus group discussions (FGD), questioners, home interviews, and personal observations have been used. The questionnaire method was used to collect information from a wide range of sources (respondents), taking into consideration their native knowledge and customs regarding the management, conservation, and use of Tef landraces in the region. In order to reach all respondents in the area, the questionnaire was first written in English and then translated into local tongues like "Afan Oromo" and "Amharic." On the basis of the preliminary survey and records acquired from district agricultural offices, the household heads were deliberately selected. In addition, all necessary genders and age groups, including senior female household heads were deliberately included to guarantee sufficient representation of the necessary diversity in indigenous knowledge.

In order to augment the data obtained from the questionnaires, interview questions were employed. In this context, semi-structured questions about the Tef landraces that are or were cultivated, their production volume in comparison to other cereal crops, production obstacles, and major uses were presented. The key informants were chosen from among the heads of households in various age groups and genders based on their practical knowledge of Tef production, conservation, and use in the area, as well as their willingness to participate in the study.

Focus group discussions with chosen Tef growing elders and experts were conducted in order to reinforce the information gathered from individual farmers and minimize missing data. The primary informants were well-known elderly farmers, 50 years of age or older, who had been active in Tef farming and conservation for more than 20 years and had lived in the area. The main topics of discussion included the estimated area being covered by Tef landraces, the existing production challenges, a list of the Tef landraces that are currently being cultivated and those that have already been lost, as well as their general opinions on the advantages of having Tef landraces in the area. Eventually, after much deliberation, a record of consolidated ideas was made.

Agricultural extension experts and development agents (DAs) from all of the selected districts and kebeles, as well as experienced researchers from Debre Zeyit Agricultural Research Center, the national center of excellence for research on Tef, were consulted to determine whether the landraces identified by local farmers were truly landraces or improved varieties. Secondary data from the Ethiopian Biodiversity Institute (EBI) and Tef researchers was also used to validate the landraces and screen the improved and unusual varieties released through the formal system.

### 2.5 Data analysis

Descriptive statistics pertaining to the respondents' socio-demographic characteristics and the challenges facing Tef landraces' current production were analyzed using Minitab version 19. Several ecological models that fit species diversity were also used to analyze the crop's levels of on-farm genetic diversity. Accordingly, each landrace was treated as a separate species and the diversity indices such as Margalef, Menhinick, Shannon-Weaver, and Simpson (equivalent of Nei) were applied [13]. Richness, or the quantity of landraces, and evenness, or the distribution of their abundance, was used to describe the diversity of the landraces. Accordingly, the richness of landraces (R) in the study districts and kebeles was determined using Margalef's (D_Mg_) and Menhinick's (D_Mn_) indices:

DMg=(L−1)/lnR;DMn=L/√R

where, L is the number of landraces in each study district or keblele; and *R* denotes the number of records for each landrace; D_Mg_ ≥ 0; D_Mn_ ≥ 0.

In a similar manner, the evenness (E) of landraces was determined as a measure of the Shannon-Weaver Information Index (GDs) using formula E = GDs/lnL where L is the total number of landraces cited in each study district or kebele; GDs is a measure of Shannon-Weaver Information Index and is given by $\text{GDs}=-\sum_{i=1}^{n}\text{PilnPi}$ ; *Pi* = the proportional abundance of the ith landraces and given by (ni/N; where ni = number of each record and N = total number of records in each district and kebele).

Sorenson's Coefficient (SC) was used to measure community similarity (landrace community, in this case) and was calculated as SC = 2C/S1+S2 where, C = the number of species (landraces) common to both districts; S1 = the total number of species (landraces) recorded in district 1; S2 = the total number of species (landraces) recorded in district 2.

The spatial diversity or abundance of landraces was ascertained using Simpson's index (D), which takes into consideration the frequency of occurrence of each recorded farmer landrace (which is thought of as a distinct species) across all districts and given as;

$$D=1-\sum_{i=1}^{n}{Pi}^{2}$$

where, *Pi*^*2*^ is the squared proportion of landrace i to the total records.

The extent of genetic erosion in Tef landraces for each district and study kebele and for the entire study area taken together was determined in terms of temporal diversity and analyzed over a period of twenty to thirty years using the formula of [14].

Accordingly, the extent (percentage) of landraces eroded over the past couple of decades was determined from the estimate of genetic integrity (GI) as GE(%) = 100 % − GI, where GE = the extents of genetic erosion; GI = the extents of genetic integrity and computed as GI(%) = (N_2_/N_1_) × 100 where N_2_ refers to number of landraces currently cultivated in the study area and N_1_ refers to the number of landraces used to be cultivated over the past twenty to thirty years.

## 3 Results and discussions

### 3.1 Results

#### 3.1.1 Demographic history of the respondents

The demographic characteristic of the respondents is presented below under Table 2. The study included 400 respondents in total; 378 (94.5 %) male and 22 (5.5 %) female. The number of male respondents was significantly greater than the number of female respondents in each of the study districts and kebeles.

**Table 2:** Proportion of the study respondents described in terms of sex, age, educational level, marital status and religion.

Study variable	Category	Shirka				Tana				Arsi Robe				Enkelo Wabe			
		Tereta	%	Hella-Zanbaba	%	Sole Chafaa	%	Bakaksa	%	Sabro Chafe	%	Ataba Robe	%	Bokoji Mirte	%	Machitu Goto	%
Sex	Male	59	**93.7**	41	**93.2**	36	**94.7**	49	**96.1**	51	**98.1**	47	**95.9**	37	**94.9**	58	**90.6**
	Female	4	**6.3**	3	**6.8**	2	**5.3**	2	**3.9**	1	**1.9**	2	**4.1**	2	**5.1**	6	**9.4**
	**Total**	**63**	**100**	**44**	**100**	**38**	**100**	**51**	**100**	**52**	**100**	**49**	**100**	**39**	**100**	**64**	**100**
Age	Less than 35	5	**7.9**	3	**6.8**	4	**10.5**	5	**9.8**	8	**15.4**	6	**12.3**	4	**10.3**	2	**3.1**
	35–45	10	**15.9**	8	**18.2**	7	**18.4**	9	**17.6**	11	**21.2**	8	**16.3**	10	**25.6**	17	**26.5**
	46–50	28	**44.5**	21	**47.7**	16	**42.2**	23	**45.1**	20	**38.4**	23	**46.9**	16	**41.0**	25	**39.1**
	Above 50	20	**31.7**	12	**27.3**	11	**28.9**	14	**27.5**	13	**25.0**	12	**24.5**	9	**23.1**	20	**31.3**
	**Total**	**63**	**100**	**44**	**100**	**38**	**100**	**51**	**100**	**52**	**100**	**49**	**100**	**39**	**100**	**64**	**100**
Education level	Only Read and write	44	**69.8**	27	**61.3**	22	**57.9**	36	**70.6**	28	**53.8**	24	**48.9**	29	**74.4**	38	**59.4**
	Primary School	17	**26.9**	15	**34.1**	12	**31.6**	11	**21.6**	16	**30.8**	19	**38.9**	8	**20.4**	19	**29.7**
	Secondary School	2	**3.3**	1	**2.3**	4	**10.5**	3	**5.9**	6	**11.5**	6	**12.2**	1	**2.6**	6	**9.4**
	College diploma and above	0	**0.0**	1	**2.3**	0	**0.0**	1	**1.9**	2	**3.9**	0	**0.0**	1	**2.6**	1	**1.5**
	**Total**	**63**	**100**	**44**	**100**	**38**	**100**	**51**	**100**	**52**	**100**	**49**	**100**	**39**	**100**	**64**	**100**
Marital status	Single	0	**0.0**	1	**2.3**	0	**0.0**	2	**3.9**	0	**0.0**	0	**0.0**	0	**0.0**	1	**1.6**
	Married	59	**93.7**	39	**88.6**	36	**94.7**	47	**92.3**	50	**96.2**	47	**96.0**	37	**94.9**	59	**92.2**
	Divorced	3	**4.8**	4	**9.1**	0	**0.0**	1	**1.9**	2	**3.8**	1	**2.0**	0	**0.0**	3	**4.7**
	Widowed	1	**1.5**	0	**0.0**	2	**5.3**	1	**1.9**	0	**0.0**	1	**2.0**	2	**5.1**	1	**1.5**
	**Total**	**63**	**100**	**44**	**100**	**38**	**100**	**51**	**100**	**52**	**100**	**49**	**100**	**39**	**100**	**64**	**100**
Religion	Muslim	49	**77.8**	29	**65.9**	23	**60.5**	37	**72.6**	30	**57.7**	29	**59.2**	21	**53.8**	38	**59.4**
	Christian	14	**22.2**	13	**29.6**	15	**39.5**	11	**21.6**	22	**42.3**	19	**38.8**	18	**46.2**	26	**40.6**
	Wakefat	0	**0.0**	2	**4.5**	0	**0.0**	2	**3.9**	0	**0.0**	1	**2.0**	0	**0.0**	0	**0.0**
	Others	0	**0.0**	0	**0.0**	0	**0.0**	1	**1.9**	0	**0.0**	0	**0.0**	0	**0.0**	0	**0.0**
	**Total**	**63**	**100**	**44**	**100**	**38**	**100**	**51**	**100**	**52**	**100**	**49**	**100**	**39**	**100**	**64**	**100**

In terms of age distribution, the majority of respondents (172, or 43.0 %) were between the ages of 46 and 50, followed by those older than 50 (111, or 27.7 %) and between 35 and 45 (80, or 20.0 %). The last share (37 or 9.3 %) was accounted for farmers with less than 35 years of age. The distribution pattern is consistent across all the study kebeles.

In terms of literacy and formal education, a greater percentage of respondents (248, or 62.0 %) could only read and write and a sizable portion (117, or 29.3 %) had only completed elementary school. The remaining smaller proportion (8.7 %) had either a secondary education or a diploma. In terms of marital status, almost all of the respondents (374 or 93.5 %) were married. Regarding religion, most of the respondent (256 or 64.0 %) farmers are Muslims while (138 or 34.5 %) are Christians.

#### 3.1.2 Tef landraces identified in the study areas and their distinctive features

Local names given to genetic resources based on indigenous knowledge are one of the markers of genetic distinctiveness. Such names are typically assigned based on the resources' unique characteristics, their special and distinctive end-use qualities, or other at least locally significant attributes [17]. As a result, recording and evaluating such data is crucial to supporting the preservation and future applications of landraces. Accordingly, in the present study, a total of 26 Tef landraces (Table 3) that are either still in production or had been cultivated in the last two to three decades were surveyed. The landraces identified by the respondents differed in terms of agronomic characteristics such as grain yield, maturity and seed color. Moreover, some were introduced by the agricultural research systems of the country before a couple of decades and stayed with the local farmers and thus constitute landraces. For example, Asgori were introduced in 1970, Magna and Wellenkomi both in 1978, Tsedey in 1984, Boset in 2012 and others were later than 2012 but earlier than 2015.

**Table 3:** List of Tef landraces cited in the study area along with their seed color, maturity, and distinctive Traits.

S/N	Landraces	Implication of the naming	Seed color	Maturity	Yields and other related characters
1	Abolse	Given by Agricultural research system	Light brown	Intermediate	Moisture stress resistance
2	Afari	To show its soil like seed color	Red	Intermediate	Moisture stress resistance
3	Aruso Tef	Refer to the indigenous Arsi clan	White	Late	High yield in optimum season
4	Asgori	Given by Agricultural research system	Brown	Early	High yield in optimum season
5	Atale	Given by Agricultural research system	Pale White	Late	Poor lodging resistance
6	Boset^a^	Well adapted to a specific area called Boset	Cream white	Early	High yield in optimum season
7	Bungn gabaaba	To refer to its short panicle	Deep red	Early	High yield in optimum season
8	Bungn dheeraa	To refer to its long panicle	Red	Intermediate	Moisture stress resistance
9	Bulga	Given by Agricultural research system	Red	Intermediate	Medium yield
10	Burssa bittinaawaa	Refer to its fairly loose panicle	Brown	Early	Low yielding
11	Burssa maramaa	Refer to its compact to semi-compact panicle	White	Early	Low yielding
12	Enatit	Given by Agricultural research system	Pale White	Intermediate	High yield in optimum season
13	Gibe	Given by Agricultural research system	White	Late	High yield in optimum season
14	Gorade	Show its short plant length	Browan	Late	High yield in optimum season
15	Kora	Qualify its high yielding and tolerance	White	Early	High yield in moisture stress
16	Korit	Suggest its less susceptibility to lodging	orange white	Intermediate	Poor lodging resistance
17	Magna	Refer to its mixed and preference for consumption	Cream white	Late	High yield in optimum season
18	Machare	Given by Agricultural research system	Cream white	Early	Medium yielding
19	Managesha	Given by Agricultural research system	White	Late	High yield in optimum season
20	Melko	Given by Agricultural research system	White	Intermediate	Moisture stress resistance
21	Mingar^b^	Refer to its adaptability at Mingar area	Cream white	Late	High yield in optimum season
22	Sergegna	Show to its mixed nature	mixed red and white	Late	Medium yielding
23	Tef Bada	Refer to its wide adaptability to highland areas	White	Late	Poor lodging resistance
24	Tikur Tef	Qualify its blackish red seed color	Red	Early	High yield in optimum season
25	Tsedey	Given by Agricultural research system	White	Early	High yield in optimum season
26	Wellenkomi	Given by Agricultural research system	Cream white	Intermediate	Moisture stress resistance

a Boset is a potential Tef growing area in East Shewa zone, Oromia region, Ethiopia.

b Mingar is one of the Tef growing area in Northern Shewa zone, Amhara Region, Ethiopia.

Regarding maturity, it has been reported that different landraces mature at different times and are therefore categorized as early maturing (two months and a few days more), medium maturing (three months and a few days more) and late maturing (four months and more). Accordingly, a considerable number of the landraces mentioned (nine of the total twenty-six such as Aruso, Atale, Gibe, Gorade, Magna, Menagesha, Mingar, Sergegna and Tef Beda) took more than four months to mature and thus belong to the late type. All the other Tef landraces belong to the early and medium types.

The Tef landraces reported in the region also had different seed colors. Most landraces had white and related colors such as pale white, cream white, orange white while all other landraces had either red, brown, light brown or mixed colors. In addition, the landraces had different characteristic traits such as differences in yield where some are remarkably higher yielding and others are medium to low yielding, differences in tolerance to extreme conditions where some are tolerant to lodging and extreme environmental conditions while others are very susceptible to lodging and extreme conditions. Some of the less-performing landraces are highly neglected in terms of cultivation and preservation.

#### 3.1.3 Extents of on-farm genetic diversity in the study area

##### 3.1.3.1 Genetic richness, spatial diversity, and evenness of the landraces

A summary of inter-landrace diversity or landrace richness (R) calculated using Margalef's (D_Mg_) and Menhinick's (D_Mn_) indices is presented below in Table 4. Overall, both indices showed higher levels of landrace richness in all the kebele and districts studied, with the smallest D_Mg_ value being 2.29 in Bakaksa kebele and 2.80 in Enkelo Wabe district; D_Mn_ being 1.40 and 1.38 in the same kebele and district, respectively.

**Table 4:** Estimates of on-farm diversity expressed in terms of Shannon Weaver index, landrace evenness (E) and richness (D_Mg_ and D_Mn_).

Districts	Kebles	No of landraces (L)	No of records (r)	Diversity indices			
				*Shannon-Weaver index (GDs)*	*Landrace evenness (E)*	*Margalef's index (D*_*Mg*_*)*	*Menhinick's index (D*_*Mn*_*)*
Shirka	Tereta	14	63	2.48	0.94	3.14	1.76
	Hella-Zanbaba	15	44	2.37	0.88	3.70	2.26
	Total^a^	16	107	2.4	0.87	3.21	1.55
Tana	Sole Chafa	14	38	2.32	0.88	3.57	2.27
	Bakaksa	10	51	2.14	0.93	2.29	1.40
	Total^a^	14	89	2.36	0.89	2.90	1.48
Arsi Robe	Sabro Chafe	13	52	2.35	0.92	3.04	1.80
	Ataba Robe	11	49	2.32	0.97	2.57	1.57
	Total^a^	15	101	2.29	0.85	3.03	1.49
Enkelo Wabe	Bokoji Mirte	13	39	2.45	0.96	3.28	2.08
	Machitu Goto	14	64	2.51	0.95	3.13	1.75
	Total^a^	14	103	2.43	0.92	2.80	1.38

a Indicates the total value for the districts considered.

As for the studied kebeles, a relatively lower estimate of landrace richness was found in Bakaksa (D_Mg_ = 2.29; D_Mn_ = 1.40) and Ataba Robe (D_Mg_ = 2.57; D_Mn_ = 1.57). On the other hand, the highest richness was found in Hella-Zenbaba (D_Mg_ = 3.70; D_Mn_ = 2.26) and Sole Chefa (D_Mg_ = 3.57; D_Mn_ = 2.27), both of which are form different districts. The kebeles also showed considerable variation (D_Mg_ = 2.29 in Bakaksa to 3.70 in Hella Zenbaba, D_Mn_ = 1.40 to 2.26 in the same districts). There weren't many differences among the three study districts considered (D_Mg_ = 2.80; D_Mn_ = 1.38 in Enkelo Wabe to D_Mg_ = 3.21; D_Mn_ = 1.55 in Shirka).

Estimate of the extent of diversity in terms of evenness index (E) (Table 4) also showed the highest value for both the kebeles studied (the smallest is 0.88 in Sole Chefa) and the districts considered (the smallest being 0.85 in Arsi Robe). Furthermore, the variation between the studied kebeles (0.88 in Sole Chefa kebeles to 0.97 in Hella Zenbaba) and districts (0.85 in Arsi Robe district to 0.92 in Enkelo Wabe district) is very small.

##### 3.1.3.2 Distribution of the landraces in the study area

The total number and distribution of the landraces in the individual kebeles and districts of the surveyed areas are shown in Table 5. Both the total number and distribution of Tef landraces recorded in each kebele and district varied, so that landraces that are popular in one kebele or district are uncommon in others and *vice versa*. On the other hand, a considerable number of landraces, e.g. Asgori, Boset, Bulga, Enatit, Gorade, Kora, Managesha, Mingar, Sergegna and Short Bunugn, were commonly mentioned in varying proportions in all the districts surveyed. Most of the above genotypes were improved varieties released by the National agricultural research system reached the area through agricultural technology extension and dissemination. Landraces like Bulga and Mingar, however, might have been brought from Shewa to Arsi areas along with people as the population movement in Ethiopia is mostly from North to South. Surprisingly, Asgori, Boset, Gorade and Sergegna were found in all the studied kebeles.

**Table 5:** Distribution and total number of the landraces in the individual kebeles and districts of the surveyed areas. “**0**” represents absence of the landraces and “**1**” represents presence.

Landraces	Kebeles		District	Kebeles		District	Kebeles		District	Kebeles		District
	Tereta	Hella Zanbaba	Shirka	Sole Chafaa	Bakaksa	Tana	Sabro Chafe	Ataba Robe	Arsi Robe	Bokoji Mirte	Machitu Goto	Honkolo Wabe
Abolse	0	0	0	1	0	1	0	0	0	0	0	0
Afari	0	0	0	0	0	0	0	0	0	0	1	1
Aruso Tef	1	1	1	0	0	0	1	1	1	0	0	0
Asgori	1	1	1	1	1	1	1	1	1	1	1	1
Atale	1	1	1	0	0	0	0	0	0	0	0	0
Boset	1	1	1	1	1	1	1	1	1	1	1	1
Short Bungn	1	1	1	1	0	1	1	1	1	1	1	1
Long Bungn	0	0	0	0	0	0	0	0	0	1	1	1
Bulga	1	1	1	1	0	1	1	0	1	1	1	1
Burssa Marema	0	0	0	0	0	0	0	0	0	1	1	1
Burssa	0	0	0	1	1	1	0	0	0	0	0	0
Enatit	0	1	1	1	1	1	0	1	1	1	1	1
Gibe	0	0	0	0	0	0	1	1	1	0	0	0
Gorade	1	1	1	1	1	1	1	1	1	1	1	1
Kora	0	1	1	1	1	1	1	1	1	1	1	1
Korit	1	1	1	0	0	0	0	0	0	0	0	0
Magna	1	1	1	1	1	1	1	1	1	0	0	0
Machare	1	1	1	0	0	0	0	0	0	0	0	0
Managesha	1	1	1	1	1	1	1	0	1	1	1	1
Melko	0	0	0	1	0	1	0	0	0	0	0	0
Mingar	1	1	1	1	1	1	1	0	1	1	1	1
Sergegna	1	1	1	1	1	1	1	1	1	1	1	1
Teffi Bada	1	0	1	0	0	0	0	0	0	0	0	0
Tikur Tef	0	0	0	0	0	0	0	0	0	1	1	1
Tsedey	0	0	0	0	0	0	1	0	1	0	0	0
Wellenkomi	0	0	0	0	0	0	1	1	1	0	0	0

**Total**	**14**	**15**	**16**	**14**	**10**	**14**	**14**	**11**	**15**	**13**	**14**	**14**

However, some landraces such as Abolse, Melko, Tseday, Afari, Atale, Bursa Merema, Korit, Mechare and Wolenkomi showed a very limited distribution with the first three restricted to only one keblele and the others to only two kebeles in a district.

##### 3.1.3.3 Genetic abundance and estimate of sorenson similarity index in the landraces

**Fig. 2:**
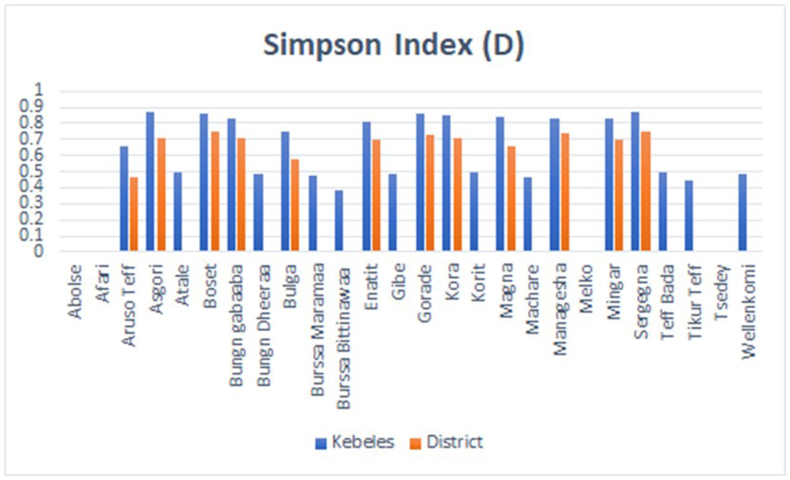
Graphical presentation of the landraces genetic abundance expressed in terms of Simpson's genetic diversity index (D) in the districts and kebeles considered.

The estimation of the genetic frequency of the landraces was expressed by the Simpson diversity index (*D*) by considering the occurrence of the landraces in one or more of the studied kebeles and possibly districts. The order of magnitude for each landrace along the studied kebeles and the respective district is shown in Table 6 and Fig. 2. Thus, the pattern of genetic frequencies in the studied kebeles and districts shows a similar pattern but with large differences in magnitude. In the studied kebeles, ten landraces, namely Asgori, Sergegna (*D* = 0.87 each), Boset, Gorade (*D* = 0.86 each), Kora (*D* = 0.85), Magna (*D* = 0.84), Bungn gabaaba, Managesha and Mingar (0.83 each) have achieved relatively high abundance. On the other hand, four landraces such as Bulga (*D* = 0.75), Aruso Tef (*D* = 0.66), Korit and Tef Bada (*D* = 0.50 each) showed moderate abundance. All the remaining landraces had relatively low abundance, ranging from *D* = 0.49 in Bungn gabaaba, Gibe and Wollenkomi to *D* = 0.00 in Abolse, Afari, Melko and Tsedey, which are confined to only one study kebele. With regards to the study districts, the landraces showed a varied abundance whereas eleven landraces showed a relatively higher estimate (*D* = 0.75 in Segegna to *D* = 0.58 in Bulga). With the exception of Aruso Tef (*D* = 0.47), all the remaining landraces showed minimal to null (*D* = 0.00) genetic abundance.

**Table 6:** Frequency and estimate of Simpson index of the landraces across the districts and kebeles considered.

Landraces	Study Kebeles and respective Districts												Total Record	Simpson Index (D)	
	Tereta	Hella Zanbaba	Shirka^2^	Sole Chafaa	Bakaksa^1^	Tana	Sabro Chafe^1^	Ataba Robe	Arsi Robe	Bokoji Mirte	Machitu Goto	Enkelo Wabe		Kebeles	District
Abolse	–	–	**-**	3	–	**3**	–	–	**-**	–	–	**-**	3	0.00	0.00
Afari	–	–	**-**	–	–	**-**	–	–	**-**	–	6	**6**	6	0.00	0.00
Aruso Tef	7	–	**7**	–	–	**-**	5	6	**11**	–	–	**-**	18	0.66	0.47
Asgori	6	5	**11**	4	6	**10**	6	4	**10**	4	3	**7**	38	0.87	0.71
Atale	3	3	**6**	–	–	**-**	–	–	**-**	–	–	**-**	6	0.50	0.00
Boset	6	3	**9**	5	4	**9**	4	3	**7**	2	4	**6**	31	0.86	0.75
Bungn gabaaba	5	2	**7**	2	–	**2**	2	3	**5**	2	5	**7**	21	0.83	0.71
Bungn Dheeraa	–	–	**-**	–	–	**-**	–	–	**-**	3	4	**7**	7	0.49	0.00
Bulga	2	1	**3**	1	–	**1**	1	–	**1**	2	5	**7**	12	0.75	0.58
Burssa Maramaa	–	–	**-**	–	–	**-**	–	–	**-**	4	6	**10**	10	0.48	0.00
Burssa Bittinawaa	–	–	**-**	1	**3**	**4**	–	–	**-**	–	–	**-**	4	0.38	0.00
Enatit	–	2	**2**	1	4	**5**	–	3	**3**	3	4	**7**	17	0.81	0.70
Gibe	–	–	**-**	–	–	**-**	3	4	**7**	–	–	**-**	7	0.49	0.00
Gorade	3	1	**4**	3	5	**8**	4	5	**9**	2	3	**5**	26	0.86	0.73
Kora	–	2	**2**	4	5	**9**	3	5	**8**	3	4	**7**	26	0.85	0.71
Korit	3	3	**6**	–	–	**-**	–	–	**-**	–	–	**-**	6	0.50	0.00
Magna	7	8	**15**	5	7	**13**	5	7	**12**	–	–	**-**	40	0.84	0.66
Machare	5	3	**8**	–	–	**-**	–	–	**-**	–	–	**-**	8	0.47	0.00
Managesha	4	2	**6**	1	6	**7**	4	–	**4**	4	4	**8**	25	0.83	0.74
Melko	–	–	**-**	2	–	**2**	–	–	**-**	–	–	**-**	2	0.00	0.00
Mingar	2	1	**3**	1	3	**4**	2	–	**2**	3	4	**7**	16	0.83	0.70
Sergegna	8	6	**14**	5	8	**13**	7	6	**13**	4	6	**10**	50	0.87	0.75
Tef Bada	2	2	**4**	–	–	**-**	–	–	**-**	–	–	**-**	4	0.50	0.00
Tikur Tef	–	–	**-**	–	–	**-**	–	–	**-**	3	6	**9**	9	0.45	0.00
Tsedey	–	–	**-**	–	–	**-**	2	–	**2**	–	–	**-**	2	0.00	0.00
Wellenkomi	–	–	**-**	–	–	**-**	4	3	**7**	–	–	**-**	7	0.49	0.00

The extent of the Sorensen's similarity index in relation to the distribution of Tef landraces in the study area is shown in Table 7. Accordingly, the pairwise similarity index revealed a higher similarity (greater than 0.50) between the districts considered. The magnitude ranged from 0.69 in Enkelo Wabe *vs* Arsi Robe to 0.77 in Shirka *vs* Arsi Robe. A similar pattern has been observed between the Kebeles considered.

**Table 7:** *Sorensen's* similarity index.

Districts	Shirka	Tena	Arsi Robe	Enkelo Wabe
**Shirka**	1			
**Tena**	0.73	1		
**Arsi Robe**	0.77	0.76	1	
**Enkelo Wabe**	0.67	0.71	0.69	1

#### 3.1.4 Temporal genetic diversity and extents of genetic erosion

Temporal genetic diversity and extents of genetic erosion in the Tef landraces considered from Arsi zone is presented below under Table 8. Consequently, of the 26 Tef landraces recalled, only 10 are grown currently, and the remaining 16 were totally eroded from the district(s) where they had previously been cultivated; as a result, the estimate of their combined genetic integrity is lower (38.46 %), with the ultimate extent of their combined genetic erosion being higher (61.54 %). However, with respect to the study districts, all had moderately higher genetic integrity (71.43 % in Enkelo Wabe to 92.86 % in Tana district) and lower genetic erosion (the highest being 28.57 % in Enkelo Wabe).

**Table 8:** List of Tef landraces unique to each study district and common to all the districts along with the extents of genetic integrity and genetic erosion.

S/N	List of Landraces lost over the past 2 to 3 decades				List of Common Landraces
	Shirka	Tana	Arsi Robe	Enkelo Wabe	
1	Atale	Abolse	Tef Aruso	Afari	Asgori
2	Korit	Bursa	Gibe	Burssa Maramaa	Boset
3	Machare	Melko	Tsedey	Bungn gababaa	Bulga
4	Tef Bada	–	Wellenkomi	Tikur Tef	Enatit
5	–	–	–	–	Gorade
6	–	–	–	–	Kora
7	–	–	–	–	Managesha
8	–	–	–	–	Mingar
9	–	–	–	–	Sergegna
10	–	–	–	–	Bunugn gababaa

No of landraces before 2 to 3 decades	16	14	15	14	26
No of landraces during 2020/21	12	11	11	10	10
Genetic integrity (GI) (%)	75.00 %	78.57 %	73.33 %	71.43 %	38.46 %
Genetic erosion (GE) (%)	25.00 %	21.43 %	26.67 %	28.57 %	61.54 %

#### 3.1.5 Major production challenges of tef landraces in the study area

The major challenges to Tef production in the study districts are presented below under Table 9. The challenges were identified and ranked in terms of their severity as highly severe (HS), medium (MS), and less severe (LS). In this regard, the respondent farmers had identified more than 11 factors as the major bottlenecks to sustainable Tef production in the area. For example, the high cost of agricultural inputs especially inorganic fertilizers has been identified as the major challenge in all the study districts. Moreover, farmers in the area largely use blanket fertilizer recommendations i. e. applying fertilizer without through soil analysis and thus, utilizing high amount on a plot of land which is sometimes resulted in lodging and high aboveground biomass without eventual high yield. Similarly, the currently increasing cost and delays in distribution have constrained the application of balanced and optimal amount of chemical fertilizers.

**Table 9:** Major Challenges/constraints to Tef production across the four Districts of Arsi Zone, Oromia Region, Ethiopia.

S/N	Challenges	Study Districts											
		Shirka			Tana			Arsi Robe			Enkelo Wabe		
		HS (%)	MS (%)	LS (%)	HS (%)	MS (%)	LS (%)	HS (%)	MS (%)	LS (%)	HS (%)	MS (%)	LS (%)
1	Poor soil fertility	66.30	27.00	6.70	90.00	10.00	–	70.50	26.00	4.50	87.00	13.00	–
2	Low yield	57.00	30.30	13.70	78.00	20.80	1.20	58.00	32.60	9.40	78.00	20.00	2.00
3	Inputs shortage	–	12.00	88.00	–	16.00	84.00	–	24.00	76.00	–	16.00	84.00
4	Lodging	48.00	22.00	30.00	48.80	30.60	20.60	46.00	27.00	27.00	52.80	28.60	18.60
5	High cost of fertilizers	76.80	18.70	4.50	91.00	9.00	–	94.80	5.20	–	93.00	7.00	–
6	Disease (leaf rust)	30.00	46.70	23.30	–	78.00	22.00	30.00	50.60	19.40	15.00	72.00	13.00
7	Weed infestation	22.30	36.70	41.00	77.00	23.00	–	45.50	17.70	36.80	89.00	11.00	–
8	Seed shattering	65.00	23.00	12.00	76.00	24.00	–	–	21.00	79.00	–	20.00	80.00
9	Threshing	30.70	32.50	36.80	28.00	36.80	35.20	20.70	32.50	46.80	25.00	31.80	43.20
10	Pest	29.70	33.10	37.20	56.00	30.00	14.00	11.70	26.10	62.20	66.00	22.00	12.00
11	Moisture stress	25.00	32.20	42.80	74.00	15.00	11.00	27.00	27.80	47.20	67.00	22.00	11.00
12	Other constraints	–	10.00	90.00	–	45.00	55.00	–	15.00	85.00	–	41.00	59.00

HS = highly severe; MS = moderately severe; LS = Less severe; Dash (−) indicates zero response.

Poor soil fertility was also reported as the major problem in Tef's sustainable production and utilization. In this regard, a larger number of the respondents (66.3 % in Shirka to 90 % in Tana districts) reported the ill effects of a reduction in soil fertility in the area because of highly pronounced soil erosion and frequent cultivation of farmlands without furrowing and appropriate shifting cultivation. Similarly, Seed shattering was also among the major Tef production constraints (65 % in Shirka to 76 % in Tana districts) and it is found to be severe especially under unexpected rainfall which is common in the region during maturity and harvest and causes heavy yield losses. Moreover, moisture stress, weed infestation, lodging, and threshing are also among the major constraints in the area.

A significant number of the respondents described that pests are also a challenge for Tef production in the study districts. Most of the farmers in the study area mentioned pests such as grasshopper (*Aiolopus longicornis*), shoot fly (different species), red worm *(Mentaxya ignicollis*), Wello bush cricket (*Decticoides brevipennis* Rag.), termites (*Macrotermes subhyalinus* and *Odontotermes* sp.) and black beetle (*Eelangeri usniger*) as the most devastating at all stages of development.

### 3.2 Discussions

#### 3.2.1 Implications of the socio-demographic history of the respondents

The socio-demographic profile of the respondents signals the importance of agriculture and Tef farming in supporting household livelihood, and how cultural and religious customs influence agricultural activities in general and Tef farming in particular. For example, the marital status of the respondent farmers (most being married) suggests the importance of agricultural activities in supporting family heads in all dimensions. It is the only means of survival for some of the respondent household farmers. Similarly, the number of male respondents being greater than females in the study area unequivocally demonstrates that women are underrepresented in agricultural practices in general and Tef production in particular. This is partially because Tef cultivation is a labor-intensive process, even though every family member participates during the sowing, weeding, harvesting, and threshing stages of the process. Moreover, the result implies that women heads are less engaged in agricultural pursuits, or even profiting from them not only in the area but also in the country which could be attributed to the deep-rooted gender-biased cultural practices.

The age of most respondents, being between 35 and 50 years old indicates that older farmers are heavily engaged in Tef cultivation and actively participating despite the labor-intensive nature of the practice. Moreover, they are valuable sources of information about the extent of genetic erosion and the primary factors contributing to the loss over the past 20–30 years. These people remembered nearly every landrace that had previously been grown in the region and were able to provide a wealth of information regarding the crop's production and use. On the other hand, the reduced involvement of respondents in the age group of under 35 suggests that there is scarce farmland in the one hand and they are not employed or engaged in other revenue-generating activities except a few on the other hand and this could increase joblessness among the vast youngsters in the area and nationwide.

Similarly, lower literacy and lack of formal education in the vast majority of the respondent farmers (the majority being less than primary school) has casted a shadow on the cultivation and preservation of the existing Tef landraces in the area. In addition, it has impacted their indigenous knowledge-driven agricultural activities and conservation of the landraces. Religious outlook has its own impact on the conservation of landraces for different end-use qualities as Tef is one of the oldest cultural and religious food sources. There is a similar report by Ref. [12] on barley landraces from Bale highlands.

#### 3.2.2 Tef landraces in the study area and implications of their naming

The indigenous knowledge-based common names given to a genetic resource are one indicator of genetic specificity, and the name usually refers to its unique distinguishing characteristics, specific or special end-use qualities that are related to cultural, religious, or commercial activities [[17], [18], [19]]. Therefore, evaluating and documenting such information is an important aspect to facilitate the preservation and further use of landraces as most are locally restricted and neglected in terms of research regardless of their rich genetic diversity and genetic variability. With this perspective, a total of 26 Tef landraces have been identified in Ethiopia's Arsi zone. The landraces differ from each other and partly from similar studies conducted so far in different parts of the country, at least in name if not genetically. The landraces differ in important agronomic traits such as maturity duration, seed color, yield, stress tolerance and end-use qualities [20,21]. Some of the landraces are still actively cultivated and are present to varying degrees in different kebeles and districts of the country. However, some have already been lost and a few are highly marginalized and on the verge of extinction.

The landraces identified in the study area are partly distinguished using their vernacular (local) names, though some were named during selection breeding by the agricultural systems in the country, which were assigned using the different characteristic features or associated features that the farmers thought important for identification and showing distinctiveness. For example, maturity length is used as one criterion to favor some landraces. In this regard, majority of the early maturing landraces are red-brown seeded with low grain yield, and highly susceptible to lodging. Beyond their color, early-maturing landraces are advantageous in the current unpredictable environmental and climatic conditions and thus, escape adverse environmental conditions during heading and flowering and grow in relatively low rainfall conditions.

On the other hand, the majority of the late maturing landraces are cream-white and white seeded with high grain yield, high biomass yield and relatively lodging tolerant, according to the respondent farmers.

The landraces also vary in seed color that was determined mainly based on the color of the seed following Quality Standard Authority of Ethiopia (QSAE) [22] standard. The colors are synonymous with their vernacular (local names) and even with their utilization. For example, a red color seeded landrace is called Afari, which was assigned to signify the color of red soil. Utilization of the mentioned landraces is largely dependent on its seed color and maturity length. For example, in most of the country's cities and rural areas, Sergegna (mixed red and white colors) is preferable over red colored. However, in the study areas, red seeded Tef landrace that have relatively short maturity duration is preferable, as similarly reported by Ref. [20]. Moreover, wide variations in seed coat colors are common across the country and it is considered as one of the key features for selection against or in favor of different end-use qualities and thus key for conservation and maintenance. There have been similar reports on the significant differences among Tef landraces in different areas or within a given area among different farmlands with regards to seed color and other morphological features [23,24].

In terms of yield and other related characteristic features, the respondent farmers indicated that each of the landraces bears their own rewarding feature that makes them preferable in the areas. For example, most of the mentioned landraces are relatively high-yielding. Similarly, some landraces are preferred for their moisture stress tolerance or being resilient to lodging which is of course, a major problem in Tef landraces across the country regardless of whether they are high-yielding or not. The result indicates that farmers rely on different rewarding characteristic features to select and maintain landraces. For example: the cream-white and white seed color fetches a high market price and thus has high market value, while the red-brown seeded landraces are preferred for their high adaptation to harsh environments such as moisture stress and poor soil fertility. There have been similar reports on Tef landraces by Refs. [20,21]. Very recently, the brown seeded Tef varieties are also getting increased global demands as well as at big restaurants in Ethiopia due to its various benefits. As a result, it becoming very common to serve both the white and brown seeded Tef injera in the hotel.

#### 3.2.3 Patterns of distribution and on-farm diversity of the landraces

The districts and kebeles considered considerably cultivate Tef landraces as compared to other cereal crops though their patterns of distribution and diversity showed slight differences so that landraces popular in one district or villages within districts seem rare in others and *vice versa*. In this regard, the higher estimates of both inter-landrace diversity or landrace richness (R) and evenness index (E) observed in all the kebeles and districts studied are partly attributed to the wide cultivation and importance of the crop in supporting the livelihood of subsistence farmers. However, such a high diversity index sometimes might not necessarily indicate the extent of genetic diversity and the possible importance of the crop since some districts may cultivate only limited Tef landrace but over a large land area that could constitute a very low diversity index. The same trend of larger pairwise similarity has been revealed by *Sorensen's* similarity index.

Similarly, the substantial number (half or 13 of the total landraces identified) of landraces scoring a higher to moderate genetic abundance (D = 0.87 in Asgori, and Sergegna to D = 0.50 in Korit and Tef Bada landraces) suggests the greater preference of Tef landraces in the area for different cultural, religious and economic benefits. The estimate could also signpost substantial within landrace diversity, (even though named differently at different districts and kebeles) because of the different farming and management practices over decades that could trigger adaptation and accumulation of genetic variations. However, yet significant number of the identified landraces are negligibly cultivated partly because of their reduced performance and less preference for local utilization and thus, bear less contribution to the livelihood of subsistence farmers. As a result, they are very close to total or partial erosion. Moreover, by its very nature and due to lack of research focus, Tef is relatively low yielding and thus there is a tendency of shifting towards commercial and relatively high-yielding crops like wheat which is currently becoming a political and food security crop gaining much attention from the local, regional, and national government.

In general, the result suggests that though Arsi zone and southeast Ethiopia are well known for wheat farming, the districts and kebeles considered are rich in Tef genetic resources and may be considered one of the centers of diversity and, thus, important sources of Tef selection and conservation. Therefore, special research and intervention actions targeting conservation and improvement of those landraces need to be put into practice beyond awakening the local community in advancing their indigenous knowledge-based utilizations.

#### 3.2.4 Patterns of temporal diversity and genetic erosion in tef landraces

The trend of temporal diversity and loss of Tef landraces in the present time is exhibiting a dramatic increase. The detectable loss in the districts considered (GE = 21.43 % in Tana to GE = 28.50 % in Enkelo Wabe) and the higher overall loss (GE = 61.53 %) in the area and eventually the country has a negative impact on the improvement and conservation of Tef germplasm beyond affecting food security issue of the area and the country at large. Such loss of previously adapted landraces is attributed to several factors such as displacement by modern, mostly uniform and high-yielding varieties, climate change-driven environmental conditions, utilization or end-use qualities, market preference, and others. For example, over the past decades, farmers have largely tilting towards using improved varieties in Arsi zone and the country at large. As a result, most of the country's landraces are being replaced by varieties from agricultural research centers [25,26]. Such preference could be attributed to getting improved productivity with good marketability or quality of grain. According to Ref. [8], higher extents of genetic erosion are a threat to further breeding and conservation.

#### 3.2.5 Tef landrace production challenges and associated consequences

Tef production and productivity is being hampered by several biotic, abiotic, and socio-economic factors, though its national average yield is gradually increasing year by year (it reached 1.92 in 2022) [27]. As a result, its national average yield is much less than other cereal crops in the country [27]. Thus, identifying and prioritizing the challenges is helpful in devising appropriate strategies and thus, enhance its production and utilization. Accordingly, the currently increasing cost of inorganic fertilizers was among the major factors which must be totally or partly replaced by cost-effective and sustainable natural organic fertilizers such as compost and manure [28]. reported the high price of inorganic fertilizers as being a major bottleneck for production and productivity improvement in Ethiopian crops including Tef landraces.

Tef production in the area is also affected by poor soil fertility which is partly attributed to the noticeable land degradation and soil erosion in the area [29,30]. had reported low soil fertility as the major constraint for crop production, especially in the highland areas of the country.

Moreover, productivity of the crop is being hampered by its inherent early shattering character which partly suggests maintenance of the ancestral allelic variations despite the rigorous domestication processes. Such variations in the landraces could be eliminated through intensive selection breeding activities [[30], [31], [32]]. have also mentioned shattering as one of the major problems of Tef production.

As compared to other cereal crops, Tef is relatively drought tolerant. However, significant yield reduction has been reported due to moisture stress in the study area and elsewhere in the country (20.5–45 %, according to Refs. [33,34], respectively). Such challenge is partly attributed to the location of the study area which is partially in the central rift valley where moisture stress is at its peak.

As compared to other cereal crops, the capacity of Tef to compete with weeds such as grass and broad-leaved, one of the main yield-limiting biotic factors, is very poor [32,35]. As a result, Tef farming demands the participation of all the household members in weed picking activities, a highly labour-intensive activity, otherwise the yield obtained would be limited. For example [36,37], reported a significant yield loss (up to 56 % and 35 %, respectively) due to weeds. The problem is prominent in the selected districts of Arsi zone. Along with weeds, pest infestation was also reported as the major constraint in the study area and the country at large [38]. However, nowadays, the problem is partly being counteracted using high frequency of tillage and recommended pesticides.

Lodging is another major problem in Tef landraces at both the study area and in the country. Its susceptibility to lodging is attributed to the weak stem and shallow root system [39,40]. According to reports, lodging is causing up to 17 % yield loss, and lowers 1000 seed weight by 35 % and rate of germination by 44 % [[41], [42], [43]]. In addition, it reduces the quality of Tef straw which is the major animal feed in the area.

Threshing is another challenge to Tef farming because of the less mechanized system of the country and the study area where all farmers harvest by hand, followed by often stacking for up to two months before threshing because of no threshing machine. As a result, the whole threshing practice is done using animal trampling, which leaves the crop on the ground and contaminates the remainder with urine and faeces that could reduce the market value [44]. reported a similar process in the country.

## 4 Conclusions

Ethiopia is one of the potential stocks of landraces including Tef which are potential genetic resources for conservation and breeding activities. Arsi is among the hotspot areas for the improvement and maintenance of Tef landraces as revealed by the present study where a large number of (26) distinct landraces have been identified. Furthermore, the estimates of on-farm genetic diversity in the landraces and the study districts suggest the rich genetic resource in the area as well as in the country and could guarantee the country's being the potential centers of origin for Tef genetic resource. However, in recent days, several biotic and abiotic factors are leading to rapid decline in cultivation and number of the existing landraces. As a result, the Tef genetic resource in the area as well as in the country is suffering from extreme genetic erosion which eventually marks a rapid loss of important agronomic traits. Likewise, artificial selection pressure imposed by local farmers for targeted end-use qualities is sidelining some of the landraces and pushing them closer to total extinction. In general, in order to exploit the maximum benefits from the crop and to meet future food needs for the world's rapidly growing human population, policymakers and researchers should pay attention to on-farm conservation and enhancement of the landraces.

## CRediT authorship contribution statement

**Fekadu Gadissa:** Validation, Software, Methodology, Formal analysis, Data curation, Conceptualization. **Muhamed Adem:** Writing – review & editing, Validation, Formal analysis, Conceptualization.

## Declaration of competing interest

The Authors have no competing interests to declare.
